# Niche convergence suggests functionality of the nocturnal fovea

**DOI:** 10.3389/fnint.2014.00061

**Published:** 2014-07-25

**Authors:** Gillian L. Moritz, Amanda D. Melin, Fred Tuh Yit Yu, Henry Bernard, Perry S. Ong, Nathaniel J. Dominy

**Affiliations:** ^1^Department of Biological Sciences, The Class of 1978 Life Sciences Center, Dartmouth CollegeHanover, NH, USA; ^2^Department of Anthropology, Washington University, St. LouisMO, USA; ^3^Research and Education Division, Zoology and EntomologyKota Kinabalu, Malaysia; ^4^Institute for Tropical Biology and Conservation, Universiti Malaysia SabahKota Kinabalu, Malaysia; ^5^Institute of Biology, University of the Philippines DilimanQuezon City, Philippines; ^6^Department of Anthropology, Dartmouth CollegeHanover, NH, USA

**Keywords:** fovea centralis, stable isotopes, *Otus lempiji*, *Otus megalotis*, *Tarsius bancanus*, *Tarsius syrichta*, diet, visual predation

## Abstract

The fovea is a declivity of the retinal surface associated with maximum visual acuity. Foveae are widespread across vertebrates, but among mammals they are restricted to haplorhine primates (tarsiers, monkeys, apes, and humans), which are primarily diurnal. Thus primates have long contributed to the view that foveae are functional adaptations to diurnality. The foveae of tarsiers, which are nocturnal, are widely interpreted as vestigial traits and therefore evidence of a diurnal ancestry. This enduring premise is central to adaptive hypotheses on the origins of anthropoid primates; however, the question of whether tarsier foveae are functionless anachronisms or nocturnal adaptations remains open. To explore this question, we compared the diets of tarsiers (*Tarsius*) and scops owls (*Otus*), taxa united by numerous anatomical homoplasies, including foveate vision. A functional interpretation of these homoplasies predicts dietary convergence. We tested this prediction by analyzing stable isotope ratios that integrate dietary information. In Borneo and the Philippines, the stable carbon isotope compositions of *Tarsius* and *Otus* were indistinguishable, whereas the stable nitrogen isotope composition of *Otus* was marginally higher than that of *Tarsius*. Our results indicate that species in both genera consumed mainly ground-dwelling prey. Taken together, our findings support a functional interpretation of the many homoplasies shared by tarsiers and scops owls, including a retinal fovea. We suggest that the fovea might function similarly in tarsiers and scops owls by calibrating the auditory localization pathway. The integration of auditory localization and visual fixation during prey detection and acquisition might be critical at low light levels.

## INTRODUCTION

The *fovea centralis*, or fovea, is an avascular declivity of the retinal surface. It is aligned with the visual axis of the eye and contains a disproportionately high density of photoreceptors. The optics of foveae are an enduring interest (Walls, 1937; Weale, 1966; Locket, 1992; Ross, 2004) because the fovea has greater spatial resolving power than other retinal specialization (Inzunza et al., 1989; Moore et al., 2012). A fovea is therefore the site of maximal visual acuity among vertebrates (Walls, 1942; Polyak, 1957; Provis et al., 2013). The energetic cost of high-acuity vision is presumed to be high due to the large volume of cortical tissue devoted to foveal vision (Perry and Cowey, 1985; Silveira et al., 1989; Hendrickson, 2005). Indeed, the tandem concept of sensory specialization and cortical overrepresentation, or magnification, is now practically idiomatic: gymnotid and mormyrid fish have electrosensory “foveas”; (Castelló et al., 2000; Bacelo et al., 2008); echolocating bats have acoustic “foveas” (Neuweiler, 2003); and some haptic species have tactile or somatosensory “foveas” (Pettigrew and Frost, 1985; Catania and Remple, 2004; Hoffmann et al., 2004; Mancini et al., 2013).

Foveal vision is assumed to serve a vital adaptive function and the comparative biology of foveate taxa has proven instructive (review: Ross, 2004). Foveae are widespread among diurnal vertebrates, but among mammals they are restricted to haplorhine primates (tarsiers, monkeys, apes, and humans). This taxonomic distribution suggests that foveae are an adaptation to diurnal or photopic conditions. The strongest support for this view stems from taxa that shifted or reversed their primary activity pattern. For example, geckos are secondarily nocturnal and a fovea is normally absent (Ross, 2004); however, some 15 genera have reverted to diurnality and regained foveate vision (Tansley, 1960; Röll, 2001). Multiple tertiary origins of foveae within Gekkonidae suggest that the selective advantages of high-acuity vision are strongest under photopic conditions. Yet some nocturnal birds and many deep-sea fish possess rod-dominant foveae (Bowmaker and Martin, 1978; Collin, 1999; Collin et al., 2000), raising the possibility that a nocturnal fovea is not always a scotopic anachronism.

**FIGURE 1:**
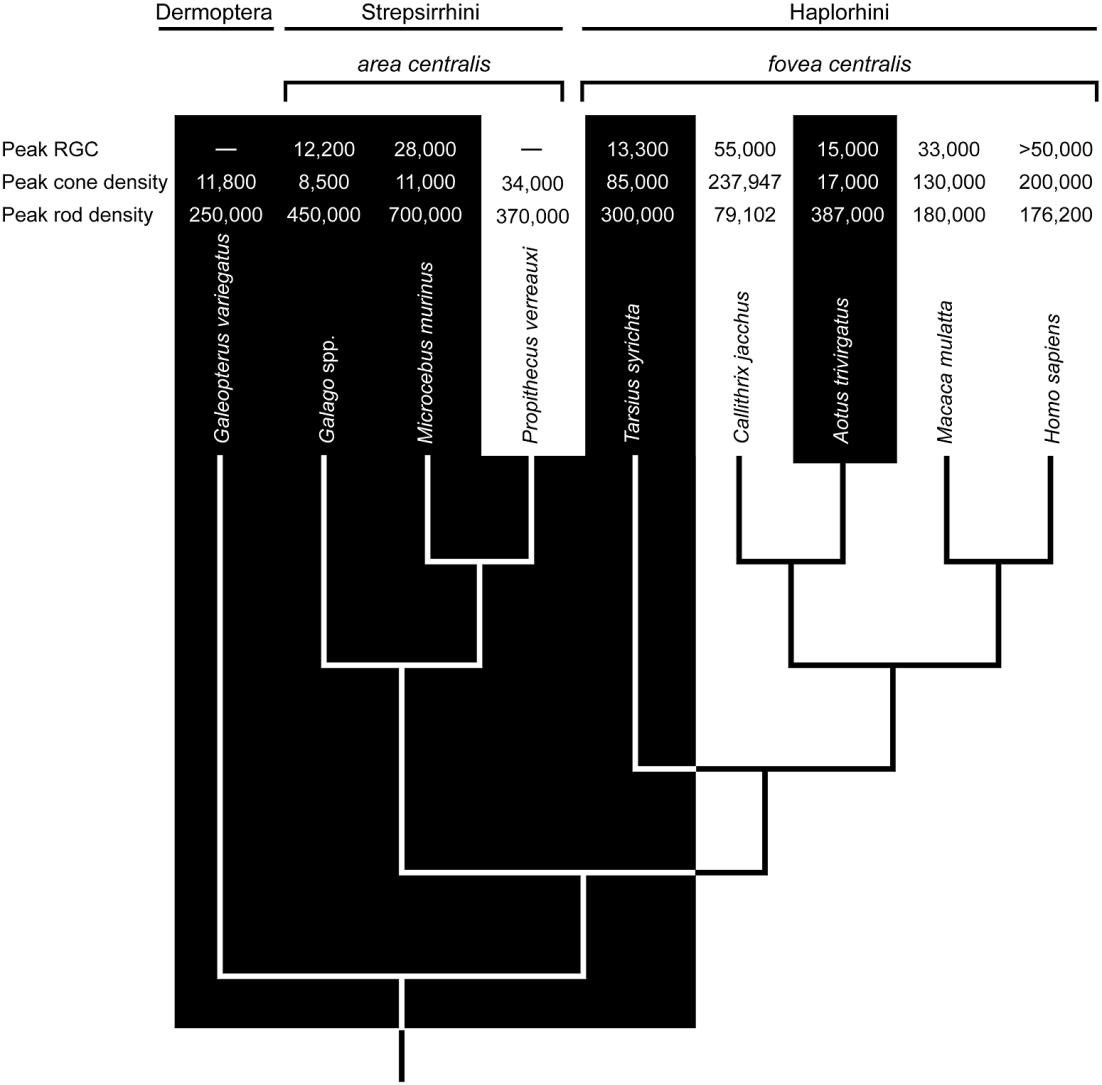
**The phyletic relationships of select primates and the sister taxon Dermoptera (the Sunda colugo, *Galeopterus variegatus*).** The distinction between nocturnal (black zone) and diurnal (white zone) activity patterns is strongly associated with variation in retinal ganglion cell (RGC) counts (mm^-2^), cone densities (mm^-2^), and rod densities (mm^-2^) in the *area centralis* or *fovea centralis* (data sources: Webb and Kaas, 1976; Perry and Cowey, 1985; Curcio et al., 1990; Wikler and Rakic, 1990; Silveira et al., 1993; Ogden, 1994; Wilder et al., 1996; Hendrickson et al., 2000; Dkhissi-Benyahya et al., 2001; Peichl et al., 2001; Ross, 2004; Tetreault et al., 2004; Finlay et al., 2008; Moritz et al., 2013). Ancestral character states based in part on these values suggest a diurnal ancestry for *Tarsius* and *Aotus*; and, by extension, stem anthropoids (e.g., Ross, 2000; Williams et al., 2010). Accordingly, the foveae of *Tarsius* and *Aotus* are most likely vestigial traits. A problem with this view is evident in the densities of RGCs, cones, and rods. Relative to *Tarsius*, the retina of *Aotus* has advanced further toward a nocturnal phenotype despite a substantially younger vintage of 5–20 million years (see text).

The question of whether nocturnal foveae are adaptations or functionless vestiges is central to the study of primate evolution. Currently, two haplorhine taxa – tarsiers (*Tarsius*) and night monkeys (*Aotus*) – are nocturnal, and the former sits at a crucial position in the primate phylogenetic tree (**Figure 1**). Tarsiers are the basal crown haplorhine primate and their fovea has long informed hypotheses on the origins of anthropoid primates (Treacher Collins, 1922; Elliot Smith, 1928; Le Gros Clark, 1959; Cartmill, 1980; Martin, 1990; Ross, 2000, 2004; Martin and Ross, 2005; Williams et al., 2010). And yet, *Aotus* has been the model taxon for understanding foveal degeneracy.

### NOCTURNAL HAPLORHINES AND THE CONCEPT OF FOVEAL DEGENERACY

The retina of *Aotus* has been studied since the 1870s (Polyak, 1957; Ogden, 1994; Silveira et al., 2001), and a rod-dominated fovea is either absent (Woollard, 1927; Detwiler, 1941; Jones, 1965; Ferraz de Oliveira and Ripps, 1968), shallow and rudimentary (Kolmer, 1930; Polyak, 1957; Wolin and Massopust, 1967; Silveira et al., 1993), or present in 10% of individuals (Ogden, 1994). Walls (1953) viewed this variation as evidence of functional degeneracy. Webb and Kaas (1976) averred, reporting a shallow fovea and displaced ganglion cells; they also suggested that a degenerate fovea is functionally comparable to an *area centralis*, the retinal specialization of strepsirrhine primates (Rohen and Castenholz, 1967; Wolin and Massopust, 1970). Indeed, the densities of rods and cones in the foveae of *Aotus azarae* and *Aotus trivirgatus* resemble those in the *area centralis* of *Galago garnetti*, a lorisid primate (Wikler and Rakic, 1990; Finlay et al., 2008). The notion of foveal degeneracy in *Aotus*, together with the absence of a *tapetum lucidum*, is widely interpreted as evidence of a diurnal ancestry, as illustrated in **Figure 1**.

A shift to nocturnality could have occurred ∼20 Ma on the basis of phylogenetic affinities with *Tremacebus*, which was plausibly nocturnal (Kay and Kirk, 2000; Kay et al., 2004; Ross et al., 2007). Recent molecular phylogenies are compatible with this view, suggesting that the stem ancestor of *Aotus* diverged from diurnal Cebidae ∼19.3 Ma (Perelman et al., 2011), whereas crown *Aotus* diversified ∼5.5 to 4.6 Ma (Menezes et al., 2010; Ruiz-García et al., 2011). Thus, the antiquity of nocturnality in the aotine lineage is between ∼5 and 20 million years. This span was evidently sufficient to favor degenerate foveae among other distinctive attributes, such as relatively enlarged eyes and orbits (Kirk, 2006; Ross and Kirk, 2007), disabling mutations of the short-wavelength-sensitive-1 (*SWS1*) opsin gene (Jacobs et al., 1996; Levenson et al., 2007), rod photoreceptors with an inverted nuclear architecture (Joffe et al., 2014), and large numbers of P retinal ganglion cells (Silveira et al., 1994) with high rod convergence to both M and P cells (Yamada et al., 2001). These traits differentiate *Aotus* from all other monkeys and are strongly convergent with nocturnal mammals; hence, the aotine visual system is almost certainly a nocturnal derivation.

The functional anatomy of the tarsier retina is more challenging to interpret (Ross, 2004). Early studies of spectral tarsiers (*Tarsius spectrum*) failed to detect a fovea (Woollard, 1925, 1926), whereas recent investigations report the uniform presence of rod-dominant, concave-sided (concaviclivate) foveae (Hendrickson et al., 2000; Hendrickson cited in Ross, 2004). Similar foveae are present in Philippine tarsiers (*Tarsius syrichta*; Polyak, 1957; Wolin and Massopust, 1967), but variable among Bornean tarsiers (*Tarsius bancanus*; Castenholz, 1965; Castenholz, 1984). On the surface, these findings point to an *Aotus*-like state of foveal degeneracy; however, the fovea of *Tarsius* is deeper, less variable, and associated with much higher cone densities (50,000–85,000 mm^-2^; Hendrickson et al., 2000; Hendrickson cited in Ross, 2004) than that of *Aotus* (5000–17,000 mm^-2^; Wikler and Rakic, 1990; Finlay et al., 2008). Another difference concerns the *SWS1* opsin gene; it is intact among tarsiers (Tan et al., 2005) and a low rate of non-synonymous to synonymous substitutions is consistent with strict purifying selection (Kawamura and Kubotera, 2004).

Modest foveal degeneracy and a functional *SWS1* opsin gene have been interpreted as evidence of a recent transition to nocturnality (Tan et al., 2005). Indeed, two recent findings support this premise. First, the rods of *T. spectrum* have a nuclear architecture that is strongly associated with diurnality (Joffe et al., 2014). Second, molecular evidence suggests that the ancestral crown tarsier possessed a cone opsin polymorphism that enabled trichromatic vision (Melin et al., 2013). The antiquity of this character trait is uncertain, with crown divergence dates ranging from ∼18.6 Ma (Springer et al., 2012) to ∼13 to 9 Ma (Melin et al., 2013), but multiple independent losses of trichromatic vision appear to have occurred in the past 5 million years (Melin et al., 2013). Such findings suggest a relatively recent history of diurnality; and yet, the fossil record is a testament to committed nocturnality. The hyperenlarged orbits of *Tarsius eoceanus* (Middle Eocene), *Tarsius sirindhornae* (Middle Miocene), and living tarsiers are most parsimoniously interpreted as evidence of continuous nocturnality for at least 45 million years (Rossie et al., 2006; Chaimanee et al., 2011). These discrepant lines of evidence are difficult to reconcile.

The foveae and rod architecture of tarsiers could be adaptations to non-photopic conditions; and, hence not necessarily vestiges of a diurnal ancestor. Melin et al. (2013) hypothesized that the hyperenlarged eyes and foveate color vision of ancestral crown tarsiers (and potentially stem tarsiers and anthropoid primates), evolved to support visual predation under dim (mesopic) light levels such as twilight or bright moonlight. These light conditions are predicted to support cone-mediated color vision (Melin et al., 2012) and favor enlarged eyes for greater visual sensitivity in the absence of a tapetum lucidum (Cartmill, 1980). This attempt at consilience is laudable but difficult to test.

### COMPARATIVE FUNCTIONAL ECOLOGY OF THE NOCTURNAL FOVEA

**FIGURE 2:**
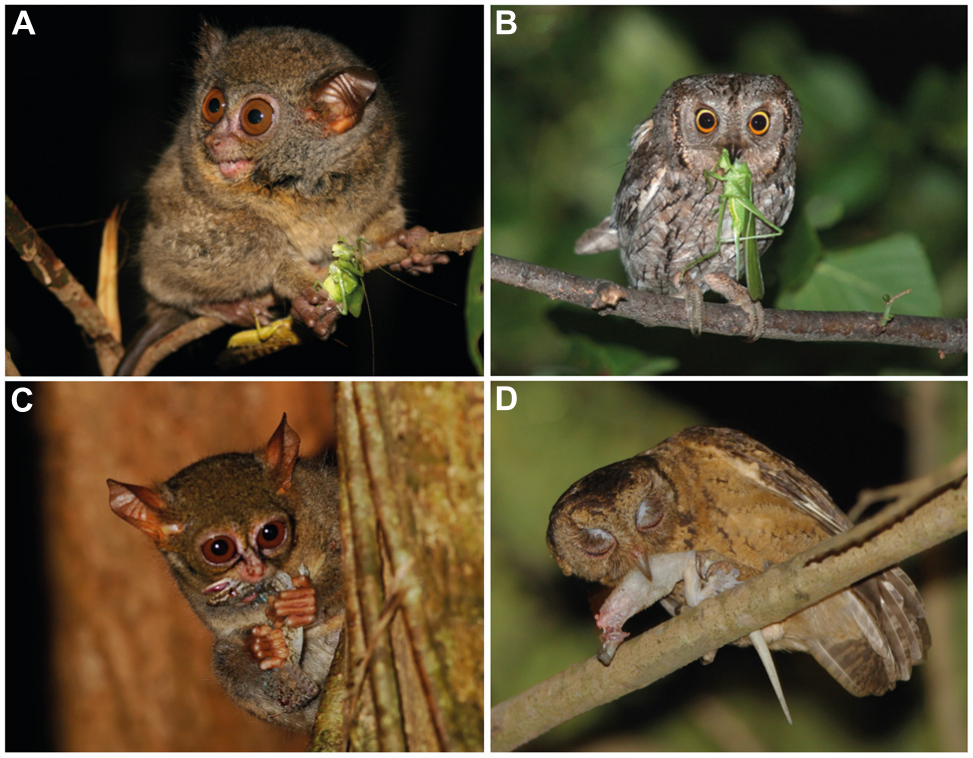
**(A)** Orthopteran insects such as katydids are a common prey item in the diet of tarsiers (photograph of *Tarsius lariang* by Stefan Merker, reproduced with permission). **(B)** Orthopteran insects are also consumed by scops owls (photograph of *Otus scops* by Clément and Julien Pappalardo, reproduced with permission). **(C)** Tarsiers also consume geckos (photograph of *T. spectrum* by David J. Slater, reproduced with permission). **(D)** In Singapore, geckos are reported to be the most common food item in the diet of *O. lempiji* (Lok et al., 2009; photograph by Tiah Khee Lee, reproduced with permission).

**FIGURE 3:**
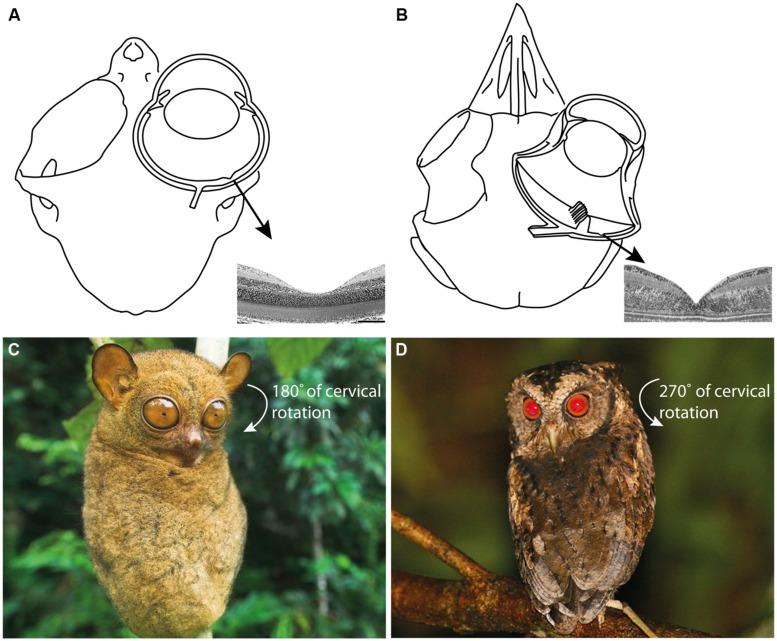
**(A)** The skull and eye of *Tarsius bancanus* (modified from Castenholz, 1984; Ross, 2004) together with the fovea of *T. spectrum* (modified from Hendrickson et al., 2000). **(B)** The skull, eye, and fovea of a composite strigiform (modified from Fite, 1973; Menegaz and Kirk, 2009). Because ocular mobility is constrained by the hyperenlarged eyes of tarsiers and scops owls, an extraordinary degree of cervical rotation is necessary to enable rapid prey localization and fixation. **(C)** The increased cervical mobility of tarsiers allows them to rotate their head 180° in azimuth (Castenholz, 1984; photograph of *T. bancanus* by Nick Garbutt, reproduced with permission). **(D)** Owls can rotate their head 270° in azimuth (Harmening and Wagner, 2011; photograph of *O. lempiji* by Paul B. Jones, reproduced with permission). Extreme head rotation is thought to enhance the sit-and-wait ambush mode of predation common to tarsiers and scops owls (Niemitz, 1985).

It is challenging for humans to observe how tarsiers discern vertebrate and invertebrate prey; they appear to integrate and alternate between auditory and visual cues depending on ambient conditions and prey type (Niemitz, 1979, 1984; MacKinnon and MacKinnon, 1980; Gursky, 2000, 2002; Dagosto et al., 2003). Such a specialized niche is assumed to have few competitors, a concept that reinforces the perception of tarsiers as “living fossils” in a state of ecological stasis (Jablonski, 2003). However, observations of the Sunda scops owl (*Otus lempiji*), a tarsier-sized faunivore (90–140 *g*), suggest a comparable niche (König and Weick, 2008; **Figure 2**). Potential niche convergence has attracted attention due to the many homoplasies that unite tarsiers and scops owls, such as (i) hyperenlarged eyes that protrude from the orbit (**Figures 3A,B**); (ii) orbit-induced displacement of the olfactory tract, which itself is unusually long; (iii) a loss of ocular mobility that corresponds with increased cervical mobility (**Figures 3C,D**); (iv) acute directional hearing; (v) enlarged semicircular canals; and, (vi) derived feeding morphologies for perforating prey (Niemitz, 1985, 2010; Menegaz and Kirk, 2009). Niemitz (1985) interpreted this suite of character traits as an adaptation to sit-and-wait ambush predation at low light levels. Evidence of dietary overlap would support this hypothesis and potentially shed light on yet another shared homoplasy, the fovea.

Within Strigiformes, there is mixed evidence for foveae in the family Tytonidae (barn and bay owls). For example, a fovea can be present (Oehme, 1961) or absent in barn owls (*Tyto alba*; Wathey and Pettigrew, 1989; Lisney et al., 2012). In the family Strigidae (“typical” owls) rod-dominant, concaviclivate foveae are uniformly present (Wood, 1917; Rochon-Duvigneaud, 1943; Oehme, 1961; Fite, 1973; Fite and Rosenfield-Wessels, 1975; Lisney et al., 2012); and at least one species, the tawny owl (*Strix aluco*), has a fovea with three cone classes (Martin and Gordon, 1974; Bowmaker and Martin, 1978). *Strix aluco* demonstrates that foveate trichromatic vision can exist in tandem with a nocturnal eye and orbit (Hall and Ross, 2007; Ross et al., 2007; Hall, 2008). Furthermore, at least two strigid species, the scops owl (*Otus scops*) and little owl (*Athene noctua*), can make chromatic discriminations at low light levels (Parejo et al., 2010; Avilés and Parejo, 2013). The retention of foveate color vision in strigids has been associated with foraging under mesopic conditions (Avilés and Parejo, 2013), a view that reinforces the possibility of tarsiers behaving similarly.

### STUDY DESIGN

A controlled experimental approach is preferable for testing foveal function; however, the mortality rate of captive tarsiers is unacceptably high (Fitch-Snyder, 2003). Accordingly, we conceived a study premised on abductive reasoning: if tarsiers and scops owls are observed to have similar diets, then the fovea that unites them can be interpreted as a functional dietary trait. A weakness of abduction is that a conclusion can remain false following verification of the initial premise. Even still, such reasoning has practical value when information is limited. Here we focus on data available in the tissues of wild-caught animals. The stable isotope ratios in these tissues can be used to quantify prior behavioral observations of dietary convergence.

Stable isotope ratios are a practical tool for quantifying the diets of difficult-to-observe animals. The isotopic niche of a species is often based on ratios of carbon (^13^C:^12^C or *δ*^13^C) and nitrogen (^15^N:^14^N or *δ*^15^N) isotopes in a two-dimensional “*δ*-space” (Newsome et al., 2007). For example, the *δ*^13^C values of animals in a savanna-woodland can vary because most plants fix atmospheric CO_2_ via two photosynthetic pathways. The *δ*^13^C values of C_3_ and C_4_ plants are ca. -28‰ (range -21 to -35‰) and -14‰ (range -12 to -16‰), respectively (O’Leary, 1988), a difference that persists in the isotopic composition of primary and secondary consumers. In a tropical forest, the isotopic baseline of plants varies to lesser extent, although factors such as canopy cover, relative humidity, light availability, tree height, and soil moisture can drive variation in *δ*^13^C values (Heaton, 1999; Amundson et al., 2003; Marshall et al., 2007). For example, C_3_ plants under sunny conditions are ^13^C-enriched (high *δ*^13^C values: -21 to -27‰), whereas those in the understory are ^13^C-depleted (low *δ*^13^C values: <-31‰) due to the recycling of CO_2_ (Kohn, 2010). This “canopy effect,” or gradient of decreasing *δ*^13^C values from the canopy to the understory (Vogel, 1978; Medina and Minchin, 1980; van der Merwe and Medina, 1991), is reflected in the isotopic composition of consumers (Schoeninger, 2010), although with a small offset due to enrichment effects. In general, the *δ*^13^C values of herbivores are 2–3‰ more positive than their diet, whereas herbivore-to-faunivore trophic enrichment can range from 0.2 to 4‰ (on the basis of keratin: DeNiro and Epstein, 1978; Roth and Hobson, 2000; Sponheimer et al., 2003; Fox-Dobbs et al., 2007; Hyodo et al., 2010; Crowley et al., 2011). Thus, *δ*^13^C values can discriminate trophic position as well as the vertical stratum of foraging within a habitat (Voigt, 2010; Rex et al., 2011), including the dipterocarp forests of southeast Asia (Kawanishi et al., 2012).

Variation in *δ*^15^N is a dietary indicator due to the systematic retention of ^15^N at each trophic level (Gannes et al., 1997). Thus increasing *δ*^15^N values are associated with trophic “steps.” A step can range from 1.3 to 5‰, but 3‰ is typical (DeNiro and Epstein, 1981; Schoeninger, 1985; Roth and Hobson, 2000; Post, 2002; Fox-Dobbs et al., 2007). In the dipterocarp forests of Borneo, the *δ*^15^N values of predators are ca. 2.6‰ higher than those of omnivores, 3‰ higher than those of herbivores, and 3.7‰ higher than those of detritivores (Hyodo et al., 2010). These results suggest that variation in *δ*^15^N can discriminate trophic levels in the habitats used by tarsiers and scops owls, although the isotopic baseline of tree leaves in northern Borneo can vary slightly as function of soil N availability (Kitayama and Iwamoto, 2001) and disturbance history (Woodcock et al., 2012). This variation is manifested in the tissues of secondary consumers. For example, Nakagawa et al. (2007) showed that the hair of omnivorous rodents in open, degraded forests were ^15^N-enriched (higher *δ*^15^N values) relative to conspecifics in primary forest, whereas *δ*^15^N values did not differ between treeshrews and squirrels inhabiting different forest types.

Thus *Tarsius* and *Otus* are predicted to have similar isotopic niches, or overlapping *δ*^13^C and *δ*^15^N values. Affirmation of this prediction would be consistent with functional interpretations of the many anatomical homoplasies shared between these two taxa, including the retinal fovea.

## MATERIALS AND METHODS

### SAMPLE ACQUISITION AND PREPARATION

**FIGURE 4:**
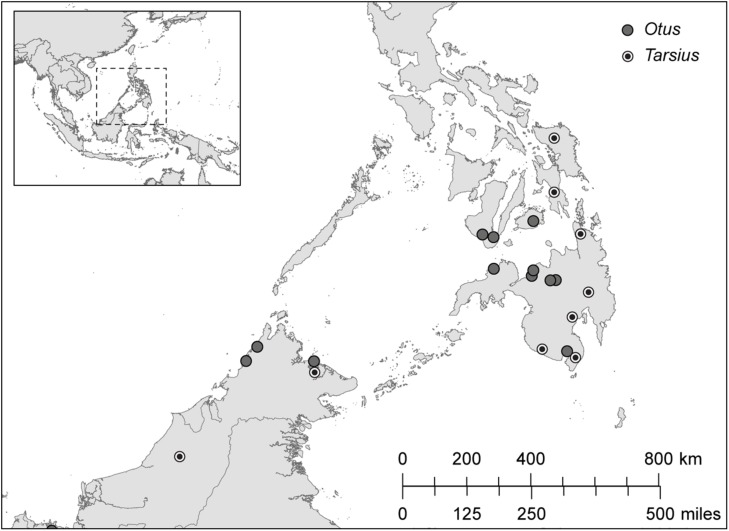
**The distribution of sampling localities in Borneo (*Otus lempiji* and *Tarsius bancanus*) and in Philippines (*O. megalotis* and *T. syrichta*)**.

We sampled the contour feathers of Sunda scops owls (*Otus lempiji*, formerly *O. bakkamoena lempiji*; *n* = 8) and Philippine scops owls (*Otus megalotis*, formerly *O. bakkamoena megalotis*; *n* = 11; taxonomy follows König and Weick, 2008). We also sampled hair from the shoulders of Bornean tarsiers (*T. bancanus*; *n* = 6) and Philippine tarsiers (*T. syrichta*; *n* = 28). The specimens, all wild-caught adults, were chosen on the basis of maximum overlapping provenience (**Figure 4**). The majority of specimens are accessioned in the American Museum of Natural History, the Field Museum of Natural History, the Kinabalu National Park Museum, and the Universiti Malaysia Sabah Museum (Appendix 1). We supplemented these samples with hair from a wild population of *T. syrichta* in the vicinity of Motorpool, Tubod, Surigao del Norte, Mindanao, Philippines (09°38′N; 125°33′E). These tarsiers (*n* = 12) were hand-caught and anaesthetized as part of a larger study of their sensory ecology (Ramsier et al., 2012). For measurements of *δ*^13^C and *δ*^15^N in keratin, 2–3 feathers or 10–15 strands of hair were cleaned of debris using ethanol, sonicated in ultrapure water, and washed 1–2 times in petroleum ether. The samples were then cut into small fragments (*∼* 1 mm) and weighed (500 ± 15 μg) into precombusted tin capsules.

### ANALYTICAL PROCEDURES

Isotope ratios are presented as *δ* values, where *δ* = 1000 ((R sample/R standard) – 1) and R = ^13^C/^12^C or R = ^15^N/^14^N; reference standards are Vienna Pee Dee Belemnite (VPDB) for carbon and atmospheric N_2_ for nitrogen. Units are expressed as parts per thousand (‰). The dried samples were combusted and analyzed with a Thermo-Chemical Elemental Analyzer (TCEA) interfaced with a Delta Plus XP isotope ratio mass spectrometer (IRMS, Thermo Finnigan, Bremen, Germany) located in the Stable Isotope Laboratory, University of California, Santa Cruz. The analytical precision (±1 SD) for *δ*^13^C and *δ*^15^N was 0.3‰ and 0.05‰, respectively, based on four International Atomic Energy Agency (IAEA) acetanilide replicates.

A potential confounding factor is associated with the steady global decrease in the ^13^C content of atmospheric CO_2_ due primarily to fossil fuel burning during the past 150 years (the Suess effect; Indermühle et al., 1999). The total magnitude of this change is ca. 1.5‰ (Long et al., 2005), but the effects within 5–10 year intervals are relatively small (ca. 0.1‰). To account for this variation in atmospheric CO_2_, which in turn is reflected in the tissues of plants and consumers, we applied conservative time-dependent correction factors of –0.004‰ or –0.02‰ per year to samples from specimens collected between 1860 and 1965 and between 1965 and 2010, respectively (Francey et al., 1999; Keeling et al., 2005).

Another confounding factor stems from geographic and temporal variation in soil N availability, both natural (Högberg, 1997; Kitayama and Iwamoto, 2001) and anthropogenic (Kendall et al., 2007; Hietz et al., 2011), and the potential for spatial autocorrelation of *δ*^15^N values. To explore this first possibility, we averaged all samples from a given site and calculated Moran’s index of spatial autocorrelation. We detected no evidence of spatial autocorrelation among sample sites (*Otus* sites: *n* = 13, Moran’s *I* = 0.23, *z* = 1.60, *p* = 0.11; *Tarsius* sites: *n* = 10, Moran’s *I* = –0.19, *z* = –0.45, *p* = 0.65), although the semivariograms are potentially uninformative due to the small number of samples spread over a relatively large spatial scale. Each *δ*^15^N value is therefore assumed to have statistical independence for assessing diet.

### STATISTICAL ANALYSES

We performed all statistical tests in R version 2.14.1 (R Development Core Team, 2011). As some of our data violated the assumptions of parametric statistical analysis, we used non-parametric Wilcoxon rank sum (two-sample) and Kruskal–Wallis *χ*^2^ (multiple comparison) tests to assess whether the carbon and nitrogen isotope compositions differentiate sympatric taxa of *Tarsius* and *Otus*. For all normally distributed data, comparisons of significance were investigated using Welch’s Two Sample *t*-tests. The significance for all tests was set at *α* = 0.05.

## RESULTS

**FIGURE 5:**
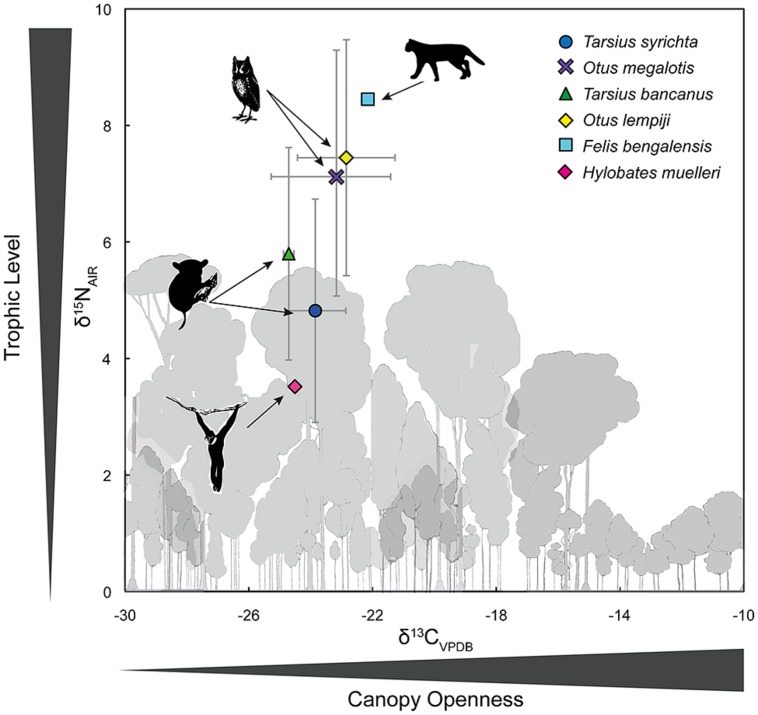
**Bivariate plot of δ^**13**^C and δ^**15**^N values (mean ± 1 SD) in the keratin of Bornean tarsiers (*Tarsius bancanus*), Philippine tarsiers (*T. syrichta*), Sunda scops owls (*Otus lempiji*), and Philippine scops owls (*O. megalotis*).** To illustrate an approximate full dietary trophic step, the keratin-derived δ^13^C and δ^15^N values of a frugivore (Müller’s Bornean gibbon, *Hylobates muelleri*) and a predator of vertebrates (leopard cat, *Felis bengalensis*) from Sabah, northern Borneo are also plotted.

Appendix 1 summarizes the raw and time-dependent corrections to *δ*^13^C. The mean ± SD of all time-corrected samples was –23.76 ± 1.6‰ (range: –27.80‰ to –17.41‰). Within Borneo, the time-corrected *δ*^13^C values of *O. lempiji* (mean: -22.87 ± 1.7‰) were ca. 1.95‰ greater than those of *T. bancanus* (mean: –24.82 ± 0.2‰), but the difference did not reach statistical significance (Wilcoxon *W* = 38; *p* = 0.08; **Figure 5**). Similarly, in the Philippines, the time-corrected *δ*^13^C values of *O. megalotis* (mean: –23.33 ± 2.5‰) were ca. 0.62‰ greater than those of *T. syrichta* (mean: –23.95 ± 1.1‰), but the difference did not reach significance (Wilcoxon *W* = 196; *p* = 0.198; **Figure 5**). Intrageneric comparisons revealed differences between the two species of *Tarsius* (*W* = 36, *p* = 0.03) but not the two species of *Otus* (*t*_16.82_ = 0.46, *p* = 0.648).

Appendix 1 summarizes the raw values *δ*^15^N. The mean ± SD of all samples was 5.79 ± 2.2‰ (range: 2.39–11.37‰). Within Borneo, the *δ*^15^N values of *O. lempiji* (mean: 7.45 ± 1.7‰) were ca. 1.65‰ greater than those of *T. bancanus* (mean: 5.80 ± 1.8‰), but the difference did not reach statistical significance (Wilcoxon *W* = 36; *p* = 0.142; **Figure 5**). The effect size of this analysis is sufficient to rule out a Type II error (Cohen’s *d* = 0.95). Within the Philippines, the *δ*^15^N values of *O. megalotis* (mean: 7.04 ± 2.3‰) were ca. 2.22‰ greater than those of *T. syrichta* (mean: 4.82 ± 1.9‰), indicating significant ^15^N-enrichment (Wilcoxon *W* = 239, *p* = 0.008; **Figure 5**); however, the samples from *T. syrichta* collected in 2010 exhibited systematically low *δ*^15^N values, perhaps due to recent anthropogenic changes to the landscape (e.g., Fox-Dobbs et al., 2012). When we calculated the mean of these samples, log-transformed all *δ*^15^N values, and controlled for specimen year in a general model, there was no statistical difference between *O. megalotis* and *T. syrichta* (*t* = –0.28, *p* = 0.781). Intrageneric comparisons revealed no differences between the two species of *Tarsius* (*t*_7.43_ = 1.19, *p* = 0.271) or the two species of *Otus* (*t*_17_ = 0.44, *p* = 0.664).

**Figure 5** also illustrates the larger food web by including the *δ*^13^C and *δ*^15^N values of a primary consumer, the frugivorous Müller’s gibbon (*Hylobates muelleri*; *n* = 1), and a predator of vertebrates, the leopard cat (*Felis bengalensis*; *n* = 1). The isotopic differences (Δ) between these taxa (Δ^13^C: 2.36‰; Δ^15^N: 4.93‰) approximate a full trophic step, albeit a rather large one. Hyodo et al. (2010) reported a similar Δ^13^C value of 2.4‰, but a smaller Δ^15^N value of 3.0‰ between herbivores and predators in Lambir National Park, Sarawak, Borneo. In any case, the magnitude of the isotopic difference (Δ) between *O. megalotis* and *T. syrichta* (Δ^15^N = 2.22) is much less than that between *Felis* and *Hylobates* (Δ^15^N = 4.93‰).

## DISCUSSION

In many respects, tarsiers are not owls, but almost (Niemitz, 2010, p. 953)

Our results demonstrate isotopic overlap: the *δ*^13^C values of *Otus* and *Tarsius* were indistinguishable, whereas the *δ*^15^N values of *Otus* were often higher than those of *Tarsius*. The low and comparable *δ*^13^C values indicate use of the same stratum (the forest floor), a foraging pattern that agrees well with behavioral observations. The differences in *δ*^15^N values – a pattern that was a trend in Borneo and temporally variable in the Philippines – are potentially instructive because they indicate a subtle degree of prey partitioning. Yet the magnitude of the isotopic difference (Δ) between *O. megalotis* and *T. syrichta* (Δ^15^N = 2.22) is much less than that between *Felis* and *Hylobates* (Δ^15^N = 4.93‰; **Figure 5**), suggesting limited partitioning of invertebrate and vertebrate prey (discussed below). However, Hyodo et al. (2010) reported a herbivore–predator Δ^15^N of 3.0‰ on the basis of a much larger data set from Sarawak. Taken together, our isotopic results demonstrate that *Otus* and *Tarsius* occupy similar dietary niches, although a trend toward ^15^N-enrichment among scops owls, particularly *O. megalotis*, suggests some prey partitioning.

For instance, it is plausible that tarsiers consume relatively few insect-eating squamates. Such an interpretation conflicts with early accounts, which stressed the central importance of geckos to the diets of *T. bancanus* and *T. syrichta* (captivity: Wharton, 1950; Harrison, 1963; wild: Fogden, 1974). However, our findings corroborate those of Davis (1962), who found a preponderance of large orthopteran insects in the stomachs of seven wild-caught Bornean tarsiers and Niemitz (1984), who observed *T. bancanus* under seminatural conditions in Sarawak. Niemitz reported that vertebrates (squamates, birds) represented <11% of 133 successful predation events. In Sulawesi, tarsiers seldom consume vertebrates (MacKinnon and MacKinnon, 1980; Tremble et al., 1993; Gursky, 2007a), but geckos can represent 4.2% of the diet (by mass) of tarsiers in captivity (Dahang et al., 2008).

A discrepancy between the foraging behaviors of wild and captive tarsiers might indicate a release from predation or competition. Perhaps in the absence of scops owls, tarsiers can shift their foraging preference to vertebrate prey. Still, recent studies of captive tarsiers in the United States report that *T. bancanus* ignores anoles (*Anolis carolinensis*) in favor of crickets, whereas *T. syrichta* exhibits the reverse pattern (Haring and Wright, 1989; Roberts and Kohn, 1993). These mixed responses to a North American anole are difficult to interpret, and they illustrate the challenge of studying the foraging adaptations of a small nocturnal visual predator. In general, our isotopic results agree well with field observations, although these are sparse – invertebrates appear to represent the great majority prey objects consumed by tarsiers.

Another possible explanation for the ^15^N-enrichment of *Otus* stems from the consumption of dung-eating (scatophagous) coleopterans:

“Food is usually sought near the ground... in villages (*Otus lempiji*) habitually hunts nocturnal insects attracted to cow dung or poultry droppings around houses. Some stomachs examined were crammed with cockroaches (Blattidae) and a particular type of black dung beetle (Scarabidae). The Sumatran (Minangklabau) name for this owl is *kuas cirit ayam*, which means ‘fowl’s-excrement owl”’ (König and Weick, 2008, p. 274).

The sobriquet “excrement owl” is potentially instructive. Animal waste is often enriched in ^15^N due to the volatilization of ^15^N-depleted ammonia, and subsequent oxidation of the residual waste material can result in nitrate with high *δ*^15^N values (Kendall et al., 2007). For example, cow dung is typically ^15^N-enriched (∼2.3‰) relative to diet (Steele and Daniel, 1978). This effect could be amplified in the dipterocarp forests of Borneo, where extended periods of protein limitation can result in ^15^N-enriched urine among large mammals, e.g., orangutans (Vogel et al., 2012). Thus, dung-eating (scatophagous) insects should be enriched in ^15^N relative to their plant-eating (phytophagous) counterparts, and the relative ^15^N-enrichment of *Otus* could reflect a greater proportion of scatophagous coleopterans or nocturnal squamates, or both, in the diet. To discriminate the relative contributions of these putative food sources, it would be useful to collect food samples and perform a Bayesian multiple source isotopic mixing model (e.g., Yeakel et al., 2009; Rutz et al., 2010).

A final possibility – that scops owls occasionally consume tarsiers – seems unlikely. Niemitz (1984) reported that *Otus* failed to induce an obvious response among Bornean tarsiers, and MacKinnon and MacKinnon (1980, p. 375) observed that spectral tarsiers “paid no attention to an owl *Ninox* sp. sitting and calling a few yards above them.” However, Gursky (2003a) reported that predator-naive infants (aged one and two months) distanced themselves from the calls of raptors (including the Sulawesi owl, *Tyto rosenbergii* and the speckled boobook, *Ninox punctulata*) and minimized movement in response to models of an ochre-bellied boobook (*Ninox ochracea*) and spotted kestrel (*Falco moluccensis*). Among adult tarsiers, the kestrel elicited the twin antipredator behaviors of mobbing and alarm calling during 47% of encounters, indicating that adults recognized it as a threat (Gursky, 2007b). The fact that no similar behaviors were directed toward owls suggests that *Otus* is an unlikely predator of *Tarsius*.

### THE TARSIER FOVEA – FUNCTIONLESS VESTIGE OR NOCTURNAL ADAPTATION?

In a report to the Zoological Society of London, the preeminent anatomist Grafton Elliot Smith described his charge to Wilfrid Le Gros Clark, who, in 1920, was appointed Principal Medical Officer to the Government of Sarawak. “I impressed upon him,” wrote Elliot Smith (1921, p. 184), “the importance of studying the retina of living or freshly-killed examples of *Tarsius*... a surviving member of the Eocene family from which our own simian ancestors were derived.” This advice from a mentor to a student rings as true today as it did a century ago; and, although the retina of *Tarsius* has since been examined in detail (Woollard, 1925, 1926, 1927; Polyak, 1957; Castenholz, 1965; Wolin and Massopust, 1967; Castenholz, 1984; Hendrickson et al., 2000; Tetreault et al., 2004), it continues to yield surprises (Joffe et al., 2014). And still, an open question remains: is the fovea a functionless vestige or a nocturnal adaptation? (Ross, 2004).

Our isotopic results are germane to this question insofar as they provide empirical evidence of food competition between scops owls and tarsiers. Although this finding entails some resource partitioning, it fails to refute the functional interpretation of the many homoplasies that unite *Otus* and *Tarsius* (Niemitz, 1985), including, very likely, the fovea. This evidence of anatomical and dietary convergence raises the possibility of parallel learning mechanisms. Perhaps a central function of the fovea is to calibrate the auditory system during development, as shown in barn owls (*T. alba*). In other words, foveate vision may guide sound localization by verifying the accuracy of auditory orientation to a sound source (Knudsen and Knudsen, 1985; Knudsen, 2002). This concept of vision-mediated or “supervised” learning (Knudsen, 1994) is compelling – Philippine tarsiers have extraordinary hearing abilities (Ramsier et al., 2012) and foveate vision could be a contributing factor to the evolution and development of their auditory localization pathway (Heffner and Heffner, 1992). Behavioral observations of tarsiers have long stressed the dual importance of auditory localization and visual fixation during prey detection and acquisition (Niemitz, 1979).

If instructed learning in the auditory localization pathway is at least partly dependent on foveate vision, then a unified representation of visual and auditory sensory stimuli was potentially a central factor in the enduring success of *Tarsius*. The initial calibration or subsequent recalibration of this system might require cone activation under non-scotopic conditions. This hypothesis could account for both the high number of cones in the fovea of *Tarisus* (relative to *Aotus*; **Figure 1**) and the phenomenon of lunar philia (increased activity under moonlight) among spectral tarsiers (Gursky, 2003b). It might also explain why the photoreceptors of tarsiers have attributes normally associated with mesopic or photopic light levels (Melin et al., 2013; Joffe et al., 2014). Taken together, the natural history of tarsiers represents a model system for studying how experience might shape the functional organization of the brain and the ensuing functional ecology of an animal.

## Conflict of Interest Statement

The authors declare that the research was conducted in the absence of any commercial or financial relationships that could be construed as a potential conflict of interest.
