# Supplementary material for: Niche convergence suggests functionality of the nocturnal fovea
Source: Front Integr Neurosci. 2014 Jul 25;8:61. doi: 10.3389/fnint.2014.00061 (PMC4110675; doi:10.3389/fnint.2014.00061)
Supplement: Supplementary file 1 [file Data_Sheet_1.DOCX]

**Appendix 1.** Source data used in the present analysis.

| **Taxon**^†^ | **Collection**^‡^ | **Accession No.** | **Island** | **Year** | **Raw δ^13^C** | **Corrected* δ^13^C** | **δ^15^N** |
| --- | --- | --- | --- | --- | --- | --- | --- |
| **Sunda scops owl** |  |  |  |  |  |  |  |
| *O. b. lempiji* | AMNH | 629855 | Borneo | 1892 | -19.28 | -20.47 | 11.37 |
|  | AMNH | 629856 | Borneo | 1892 | -21.74 | -22.93 | 7.33 |
|  | AMNH | 629858 | Borneo | 1887 | -23.72 | -24.93 | 6.31 |
|  | AMNH | 629859 | Borneo | 1887 | -20.65 | -21.86 | 6.77 |
|  | AMNH | 629860 | Borneo | 1888 | -21.19 | -22.4 | 6.41 |
|  | AMNH | 629861 | Borneo | 1886 | -22.25 | -23.47 | 7.85 |
|  | FMNH | 100782 | Borneo | - | -21.38 | -21.38 | 6.5 |
|  | FMNH | 211756 | Borneo | 1950 | -24.59 | -25.55 | 7.03 |
| **Philippine scops owl** |  |  |  |  |  |  |  |
| *O. bakkamoena* | AMNH | 802463 | Luzon | 1970 | -16.61 | -17.41 | 10.26 |
|  | FMNH | 184104 | Mindanao | 1947 | -21.56 | -22.53 | 10.26 |
|  | FMNH | 224489 | Bohol | 1955 | -21.04 | -21.89 | 10.41 |
| *O. b. everetti* | FMNH | 215141 | Mindanao | 1952 | -22.22 | -23.17 | 6.48 |
|  | FMNH | 283803 | Mindanao | 1968 | -22.88 | -23.72 | 6.32 |
|  | FMNH | 283804 | Mindanao | 1968 | -24.56 | -25.4 | 4.88 |
|  | FMNH | 357431 | Mindanao | 1992 | -23.68 | -24.04 | 5.25 |
|  | FMNH | 357432 | Mindanao | 1992 | -27.44 | -27.8 | 6.72 |
|  | FMNH | 357433 | Mindanao | 1992 | -23.62 | -23.98 | 3.52 |
| *O. b. nigrorum* | FMNH | 191232 | Negros | 1949 | -22.14 | -23.11 | 5.87 |
|  | FMNH | 209805 | Negros | 1950 | -22.49 | -23.45 | 7.46 |
| **Bornean tarsier** |  |  |  |  |  |  |  |
| *T. bancanus* | AMNH | 106010 | Borneo | 1935 | -23.73 | -24.75 | 3.31 |
|  | AMNH | 106754 | Borneo | 1937 | -24.08 | -25.1 | 4.25 |
|  | FMNH | 76858 | Borneo | 1950 | -23.92 | -24.88 | 6.37 |
|  | FMNH | 76862 | Borneo | 1950 | -23.83 | -24.79 | 8.3 |
|  | FMNH | 76863 | Borneo | 1950 | -24 | -24.96 | 6.99 |
|  | UMSM | 52 | Borneo | - | -24.49 | -24.49 | 5.58 |
| **Philippine tarsier** |  |  |  |  |  |  |  |
| *T. syrichta* | AMNH | 166856 | Mindanao | 1947 | -22.86 | -23.83 | 7.06 |
|  | AMNH | 187935 | Leyte | 1961 | -21.35 | -22.26 | 4.85 |
|  | AMNH | 203296 | Philippine Is | 1962 | -23.14 | -24.06 | 5.09 |
|  | AMNH | 203297 | Philippine Is | 1962 | -22.72 | -23.63 | 5.29 |
|  | AMNH | 207006 | Negros | 1963 | -20.74 | -21.65 | 5.2 |
|  | AMNH | 242091 | Mindanao | 1924 | -21.95 | -23.01 | 5.59 |
|  | FMNH | 56159 | Mindanao | 1946 | -22.19 | -23.17 | 4.23 |
|  | FMNH | 56740 | Mindanao | 1947 | -22.66 | -23.63 | 5.76 |
|  | FMNH | 56756 | Mindanao | 1947 | -23.22 | -24.19 | 8.15 |
|  | FMNH | 56757 | Mindanao | 1947 | -22.77 | -23.74 | 5.11 |
|  | FMNH | 56759 | Mindanao | 1947 | -23.24 | -24.22 | 5.09 |
|  | FMNH | 56763 | Mindanao | 1947 | -22.12 | -23.09 | 7.04 |
| FMNH  FMNH | 56771  61469 | Mindanao  Mindanao | 1947  1946 | -22.53  -22.99 | -23.5 | 10.32 |  |
|  |  |  |  |  | -23.96 | 6.82 |  |
| FMNH | 87715 | Samar | 1957 | -19.88 | -20.82 | 4.36 |  |
|  | FMNH | 87716 | Samar | 1957 | -22.78 | -23.72 | 6.37 |
|  | GLM | 1 | Mindanao | 2010 | -24.91 | -24.91 | 2.93 |
|  | GLM | 2 | Mindanao | 2010 | -24.29 | -24.29 | 2.69 |
|  | GLM | 3 | Mindanao | 2010 | -24.63 | -24.63 | 3.13 |
|  | GLM | 4 | Mindanao | 2010 | -24.67 | -24.67 | 3.74 |
|  | GLM | 5 | Mindanao | 2010 | -24.87 | -24.87 | 3.56 |
|  | GLM | 6 | Mindanao | 2010 | -24.84 | -24.84 | 3.66 |
|  | GLM | 7 | Mindanao | 2010 | -24.95 | -24.95 | 3.21 |
|  | GLM | 8 | Mindanao | 2010 | -24.42 | -25.42 | 3.76 |
|  | GLM | 9 | Mindanao | 2010 | -24.81 | -24.81 | 4.14 |
|  | GLM | 10 | Mindanao | 2010 | -24.88 | -24.88 | 2.39 |
|  | GLM | 11 | Mindanao | 2010 | -25.03 | -25.03 | 2.67 |
|  | GLM | 12 | Mindanao | 2010 | -24.82 | -24.82 | 2.76 |

^†^Here we report the taxonomic classifications of each museum collection. The current taxonomy of scops owls is in a state of flux; and, in the case of the Sunda scops owl, the subspecific classification has been elevated to species status. Hence, *Otus* *bakkamoena* *lempiji* is currently *O.* *lempiji.* In the case of the Philippine scops owl, the species nomen *O. bakkamoena* has been superseded by *O. megalotis*. In the main text, we use this revised nomenclature, which follows König and Weick (2008).

^‡^ AMNH=American Museum of Natural History; FMNH = Field Museum of Natural History; GLM = G.L. Moritz Personal Collection; UMSM = Universiti Malaysia Sabah Museum

*Time-corrected δ^13^C values are referenced to 2010.
